# Miniature Compressive Ultra-spectral Imaging System Utilizing a Single Liquid Crystal Phase Retarder

**DOI:** 10.1038/srep23524

**Published:** 2016-03-23

**Authors:** Isaac August, Yaniv Oiknine, Marwan AbuLeil, Ibrahim Abdulhalim, Adrian Stern

**Affiliations:** 1 Department of Electro-Optical Engineering, Ben-Gurion University of the Negev, P.O.B. 653 Beer-Sheva 8410501, Israel; 2 The Ilse Katz Institute for Nanoscale Science and Technology, Ben-Gurion University of the Negev, P.O.B. 653 Beer-Sheva 8410501, Israel

## Abstract

Spectroscopic imaging has been proved to be an effective tool for many applications in a variety of fields, such as biology, medicine, agriculture, remote sensing and industrial process inspection. However, due to the demand for high spectral and spatial resolution it became extremely challenging to design and implement such systems in a miniaturized and cost effective manner. Using a Compressive Sensing (CS) setup based on a single variable Liquid Crystal (LC) retarder and a sensor array, we present an innovative Miniature Ultra-Spectral Imaging (MUSI) system. The LC retarder acts as a compact wide band spectral modulator. Within the framework of CS, a sequence of spectrally modulated images is used to recover ultra-spectral image cubes. Using the presented compressive MUSI system, we demonstrate the reconstruction of gigapixel spatio-spectral image cubes from spectral scanning shots numbering an order of magnitude less than would be required using conventional systems.

Spectroscopic imaging generally requires an illumination source and a spectral light measurement system. With conventional spectroscopic imaging systems the scene is illuminated by a uniform light field that has a broad spectral composition and the reflected light is measured with a spectrally sensitive system. The most common approach for capturing the spatial and spectral information is first to split the spectral components, followed by direct measurements of each component. Such an approach can be implemented using spectral-to-spatial mapping based on diffractive or dispersive optical elements. Alternatively, spectral filtering can be achieved using tunable narrow band spectral filters placed within the imager.

In contrast to implementations based on direct measurements, there are sensing systems that make use of indirect, multiplexed and coded measurements for spectroscopy. The use of multiplexed, or coded, measurements for spectroscopy is not new; multiplexed spectral measurements were already in use at the advent of modern spectroscopy. Well known examples of multiplexed spectroscopy are infrared Fourier transform spectroscopy^1^ and Hadamard coding spectroscopy^2^. The use of multiplexed measurements for spectroscopy offers some advantages^3^,^4^,^5^ over the use of direct measurements, but at the cost of less intuitive system design and the need for post-processing. The matrices used to model the coded systems are essentially different from those of direct measurement systems. The system sensing matrix for the indirect measurement process must exhibit a non-zero off diagonal elements structure. This means that each single measurement provides information carried by multiple wavelengths with different weights. In general, the sensing matrix is chosen to be orthogonal, or at least non-singular, with *n* columns and *n* rows. Both direct and indirect conventional measurement systems require a number of measured points, *m*, greater than or equal to the number of points in the hypercube grid, *n*. Here a spectrometric imaging method is presented that requires significantly less measurements, *m*, than the number of points, *n*, in the hypercube grid. It uses a compact, cost effective, single LC variable retarder. This is accomplished by taking advantage of the framework of CS theory^6^,^7^,^8^,^9^. Compressive sensing theory postulates that, given some technical constraints on the sensing system and on the signal to be measured, it is possible to reconstruct a signal with *n* points from a smaller number, *m* < *n*, of measurements, using appropriate reconstruction algorithms. According to CS theory, the sensing matrix representing the measurement system can be in a non-square form of size *m* × *n* with *m* < *n* and, therefore, non-invertible. Recently, an increasing effort has been made to implement CS theory in the fields of imaging and spectroscopy^10^,^11^,^12^,^13^,^14^,^15^,^16^. The use of CS for spectrometric imaging is primarily motivated by the need to work with a very large amount of data with inherent compressibility or sparsity^17^. The common approach adopted in CS spectrometric imaging design employs indirect spectral encoding by utilizing optical spectral-to-spatial transformations.

In this paper a novel concept for spectroscopic imaging is outlined, in which the spectral encoding is accomplished entirely in the spectral domain, and no spectral-to-spatial transformations are required. Thus the system volume can be dramatically reduced. The spectral modulator is schematically presented in Fig. 1. Basically, it is composed of a specially designed Liquid Crystal Cell (LCC) attached to a photo sensor array. The LCC is designed to work as a spectral modulator that is compliant with CS theory. Therefore, the entire spectral sensor device (Fig. 1) can be built with a width of only a few millimeters. Hence, the presented approach has the advantage of compactness when compared to the more bulky methods of spectral measurement with Lyot-Öhman^18^ or Šolc^19^ spectral filters, as well as with the Fabry-Perot etalon^20^ spectral filter. These classical methods are based on direct spectral measurement^18^,^19^,^20^ implemented by narrow band spectral scanning. In addition, the physical implementation of the Lyot-Öhman and Šolc optical filters is, in most cases, by means of a set of a few cascaded spectral filters that are optically less efficient.

Using our LCC modulator^21^, *m* spectrally multiplexed images are taken and, in accordance with CS theory, from those *m* < *n* images the *n* spectral bands can be recovered by appropriate algorithms. An alternative imaging process, sharing a similar mathematical model, comprises using our LCC modulator to generate *m* spectrally structured illumination patterns and measuring the corresponding *m* intensity maps of the transmitted or reflected light. The key element in both measuring approaches is the LCC, which operates as a compact spectral encoder. By changing the applied voltage over the LCC, the effective refractive index can be controlled. The tunability of the refractive index provides a way to add different phase delays for different wavelengths and leads to wavelength dependent attenuation. The LCC is designed to have a relatively thick cavity to facilitate modulation over a broad spectrum with oscillatory behavior, as can been seen in the system matrix shown in Fig. 2. Yet the LCC cavity is still in the order of tens of micrometers thus the system in Fig. 1 has a small form factor. Liquid crystal display technology is now mature and there is a growing interest in its non-display photonic applications^22^,^23^,^24^. The system spectral encoding matrix represents the spectral encoding of the system as a function of the voltage applied. The spectrally modulated light passing through the LCC is integrated at each pixel in the sensor array. The process is in parallel over a set of pixels, since each pixel is similarly spectral encoded. This means that the spatial and spectral sensing processes are separate; therefore the entire spatial-spectral system sensing matrix can be modeled using a Kronecker product between a unit matrix of spatial mapping and the set of sensing lines that were used for the spectral encoding process (see Methods). The use of the set of *m* captured images with the measured system sensing matrix in the CS framework allows numerical reconstruction of *m* ≪ *n* images that are associated with *n* spectral bands in a miniature size.

## Results

**Figure 1:**
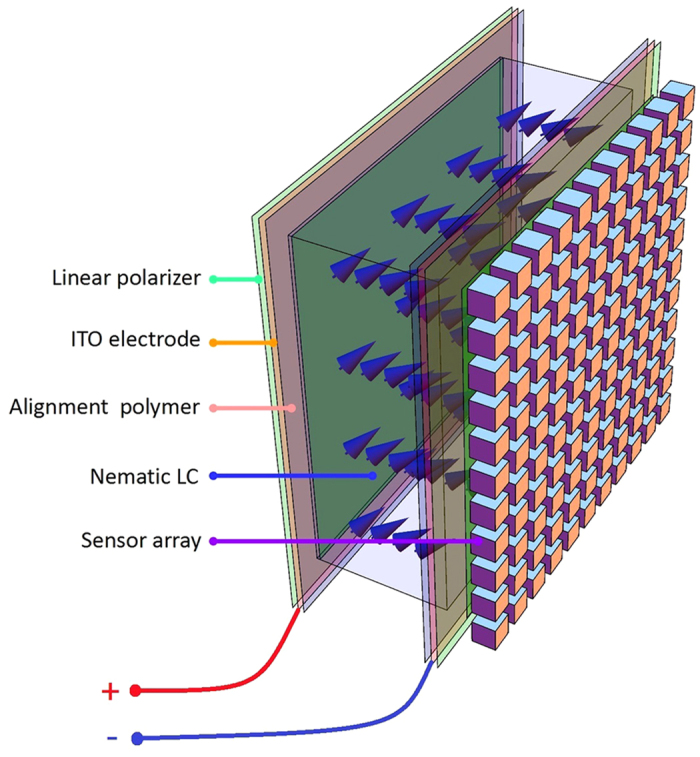
The compact ultra-spectral sensor structure. The spectral modulator imaging device comprises a grayscale standard imaging array sensor (purple array) attached to a relatively thick (~order of tens micrometers thick with clear aperture size of about a centimeter) LC phase retarder. The phase retarder is made of a LC layer sandwiched between two flat glass plates and two linear polarizers (green layers). The glass plates are coated with Indium Tin Oxide (ITO, orange layers) and a polymer alignment layer (pink layers). The cavity is filled with a commercially available LC material E44 (Merck) but selected to have high birefringence and fast enough. The birefringence is controlled by an electric field, where a voltage is applied on the ITO electrodes. In this figure, the anti-parallel alignment of the LC molecules (marked in blue) is modified as a function of the applied voltage, changing the effective retardance.

**Figure 2:**
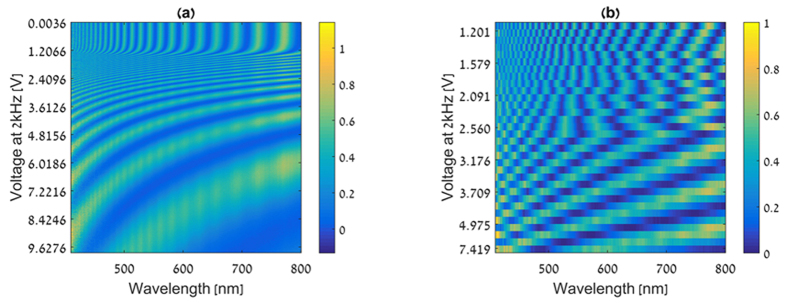
The spectral response map and system sensing matrix. (**a**) Spectral response map measured in the calibration process. Each row represents the spectral transmission of the entire system as measured for a given LCC operating voltage. It can be seen that high birefringence sensitivity exists in the range of 1 to 9 V. (**b**) Sensing matrix map used during a spectroscopic imaging with 32 random rows from (**a**).

The CS-MUSI system performance was demonstrated by building an experimental electro-optical setup with an equivalent modulator, as shown in Fig. 1 (see Methods - Experimental setup). Prior to the experiments the system was calibrated, as described in the Methods – Calibration section. In the calibration, the spectral response of the system was measured for a large set of LCC operation voltages. The spectral response map is given in Fig. 2a.

In the compressive spectroscopic imaging process, a small random subset of the operating voltages was used. From the spectral response calibration map (Fig. 2a), the corresponding spectral responses of the chosen set of operation voltages defines the sensing matrix (Fig. 2b). The set of operation voltages used is built by a random selection process with a non-uniform sampling density due to the non-uniform birefringence sensitivity of the LC to the operational voltage (Fig. 2a). For example, voltages below approximately 1 V and voltages above 9 V do not cause any change in the birefringence, therefore in each of this ranges only one voltage is chosen randomly. The operational voltages were picked randomly according to the sequential forward floating selection method^25^.

### Emission spectroscopic imaging

**Figure 3:**
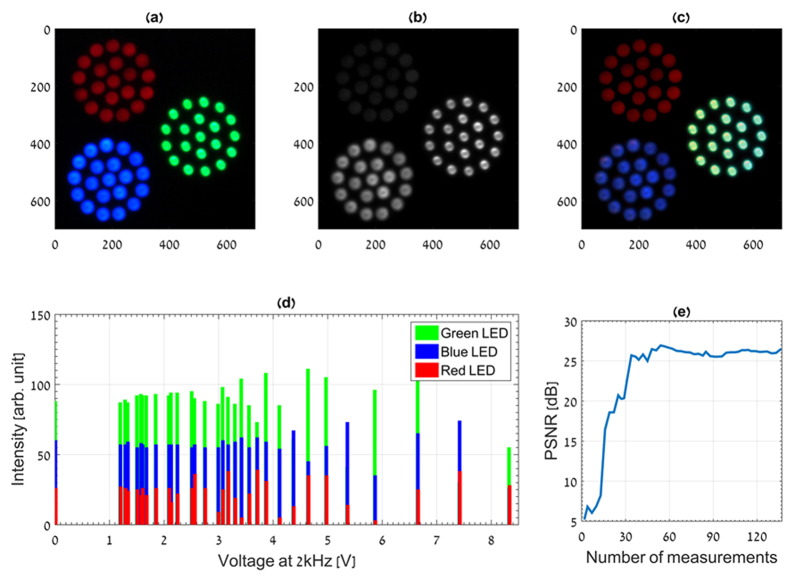
Hyperspectral imaging of LED light sources of different colors. (**a**) RGB color image of three LED arrays used as objects to be imaged with CS-MUSI, (**b**) representaive single exposure image (LCC voltage of 4.973 V) from the experimental data set of 32 images, (**c**) pseudo-color image (CIE 1964)^34^ generated from the 0.2 gigavoxel hyperspectral reconstructed data cube, (**d**) encoded measured spectral data for three object points of different colors. (**e**) Reconstruction PSNR versus the number of measurements.

First the CS-MUSI system performance is demonstrated in an emission imaging spectroscopy scenario. Two experiments are presented, the first using wide spectral distribution light sources (three color LED array) and the second with a narrow spectral distribution light sources (red and green laser sources). The first experiment was made by measuring the emission spectra of three arrays of red, green and blue light sources (Thorlabs LIU001, LIU002 and LIU003 LED array). The spectral power density of the LEDs source array was measured in the calibration process and it was found to be 470 ± 16 nm, 515 ± 21 nm, 658 ± 12 nm (FWHM) for the blue, green and red LEDs arrays, respectively. Figure 3a shows the image of the light sources captured by a standard RGB color camera. Figure 3b shows one of the captured images that represents a single grayscale frame from the compressed data set. The image shows the total optical intensity that has passed through the phase retarder and was collected by the sensor array at a given shot with a given LCC operating voltage. Due to the spectral multiplexing, the image in Fig. 3b cannot be associated with a specific wavelength. To recover the spectral information, the entire set of images needs to be used with an appropriate reconstruction process and choice of sparsifing domain. The choice of appropriate sparsifier domain depends on the mutual coherence of the sensing matrix and the compressibility of the spectral signal.

The spectral encoding of the system is changed as a function of the applied voltage over the LCC, and spectrally multiplexed measurements are captured. Figure 3d demonstrates the multiplexed intensity measurement as a function of the operation voltage for three points in the scene. Those bar graphs show the measured optical intensity of the compressed data for three different object points of different RGB colors as a function of the voltage applied on the LCC. Each bar in the graph shows the intensity value, on a scale of 256 grayscale levels, as captured by the appropriate pixel in the image plane. In terms of CS, each measurement at each location (e.g. each point in Fig. 3d) represents the results of an inner product between the spectral signal distribution and the sensing matrix, represented by the respective rows in Fig. 2b^21^. In addition, Fig. 3d also demonstrates non-uniform random selection of the operation voltages. Figure 3e shows the reconstruction peak signal-to-noise ratio (PSNR) dependence on the number of measurements. It can be seen that above approximately 30 measurements, the quality of the reconstruction in term of PSNR is approximately constant. Accordingly the hyperspectral cube was captured by taking only 32 images of size 1024 × 1280 pixels. The images were captured and then a central block of 700 × 700 pixels was cropped. Using the TwIST^26^ solver a hyperspectral cube with 391 spectral bands was restored. Figure 3c shows a pseudo-color image obtained by projecting the hyperspectral cube onto the RGB color space.

**Figure 4:**
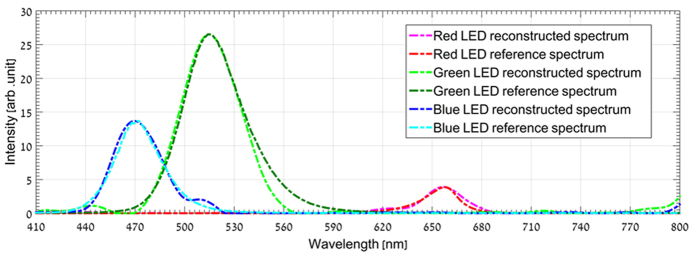
Comparison between spectra reconstructed using the proposed approach and those measured with a spectrometer. Comparison between the reconstructed spectra of three different color LEDs points and their spectroscopic measurements with a conventional spectrometer system. The three dashed line curves represent the system reconstruction signal and the line curves represent the LEDs spectral distribution as measured with a grating spectrometer.

Figure 4 demonstrates spectrum reconstruction for three points in the hyperspectral cube and their comparison to the measured spectrum of the three respective LEDs with a commercial grating based spectrometer.

The sparsifying transform that was used for reconstructing the spectral information in Fig. 4 was orthogonal Daubechies-9 wavelet. The reconstruction PSNR was 32.4 dB, 28.8 dB and 27.9 dB for the blue, green and red LEDs points, respectively.

**Figure 5:**
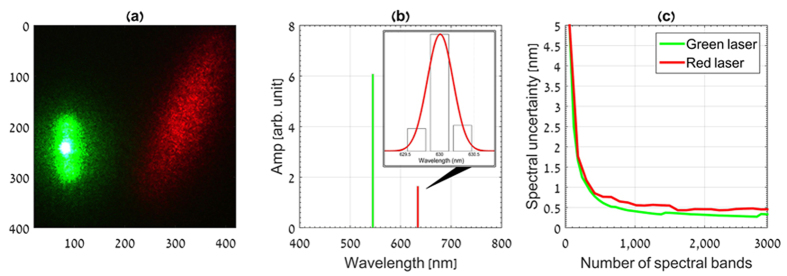
Ultra-spectral imaging of red and green lasers. (**a**) Color image of two lasers reflected from a lambertian surface. (**b**) Spectral recovery of green and red lasers from *m* = 32 exposures at *n*_*λ*_ ≈ 1000 spectral bands. (**c**) Spectral uncertainty graph. The graph shows the system spectral uncertainty as a function of the number of spectral bands.

The second experiment was made by measuring the emission spectra of two laser diodes (green and red). The experiment was conducted with the same optical setup but by replacing the LEDs array with a Lambertian surface that reflects the light from the two lasers. Figure 5a shows a cropped image of the lasers spots captured by a standard RGB color camera. As in the LEDs experiment, a set of 32 measurements images were captured. No sparsifier operator was used in the reconstruction process because in this case the spectrum signal is sparse in the spectral domain. Figure 5b shows the recovered spectrum clearly indicating the two spectral lines of the two lasers.

Since the lasers can be considered spectral impulses, the width of the reconstructed laser lines can be considered as the spectral resolution of the MUSI-sensor system and we can use this number to determine the optimal spectral division. The laser spectral lines were reconstructed with a variable division over the spectral range. Figure 5c presents the spectral uncertainty (i.e., reconstructed line width) as a function of the number of reconstructed spectral bands. It can be seen that for low number of spectral bands there is high spectral uncertainty due to the coarse spectral division. Improvement is achieved by increasing the number of bands (yielding finer spectral division) up to approximately 1000 spectral bands. Above *n*_*λ*_ ≈ 1000 no further improvement is obtained and the spectral uncertainty is approximately constant with a value of 0.4 nm for the green laser line and 0.48 nm for the red laser line.

### Reflection spectroscopic imaging

**Figure 6:**
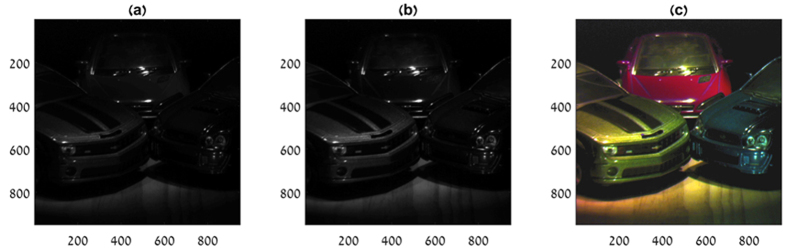
Ultra-spectral imaging of three car models: (**a**) single exposure image from the experiment with no voltage applied on the LCC. Each pixel represents a spectral multiplexed measurement and hence the image cannot be associated with a specific color or spectral band, (**b**) image captured with almost saturated LCC and therefore with neglible birefringence, (**c**) image generated by projecting the reconstructed ultra-spectral data cube onto the standard RGB color space.

Figure 5a,b show results of the ultra-spectral imaging of light reflected from extended objects. In this experiment, the illumination source was a Halogen light and the reflected light from three car models was captured by the CS-MUSI system. This experiment demonstrates the spatial imaging performance of the system. Owing to the separability of the spatial and spectral behavior of the acquisition process (see Methods), the spatial resolution is not degraded by the spectral sensing process. Figure 6a,b demonstrate two compressive images that were captured by the system. Figure 6a was captured by operating the LCC at zero voltage. Low operation voltage causes high birefringence, yielding the spectral encoding behavior described in the upper row of Fig. 2a. Figure 6b was captured with a LCC voltage of 9.7936 Vrms. At this voltage the LCC device is near its saturation state and its birefringence is very low. This case is similar to a system that is operated without the LCC device, e.g. a regular grayscale camera. It can be seen that the spatial images in Fig. 6a,b are very similar, owing to the fact that the encoding is performed only in the spectral domain and there is no spatial encoding.

Figure 6c presents a pseudo-color image obtained by projecting the reconstruction ultra-spectral cube onto the RGB space.

The image in Fig. 6c appears to have the same quality as those in Fig. 6a,b. The fine spatial details of the car model are preserved in the imaging process and no spatial degradation can be observed.

**Figure 7:**
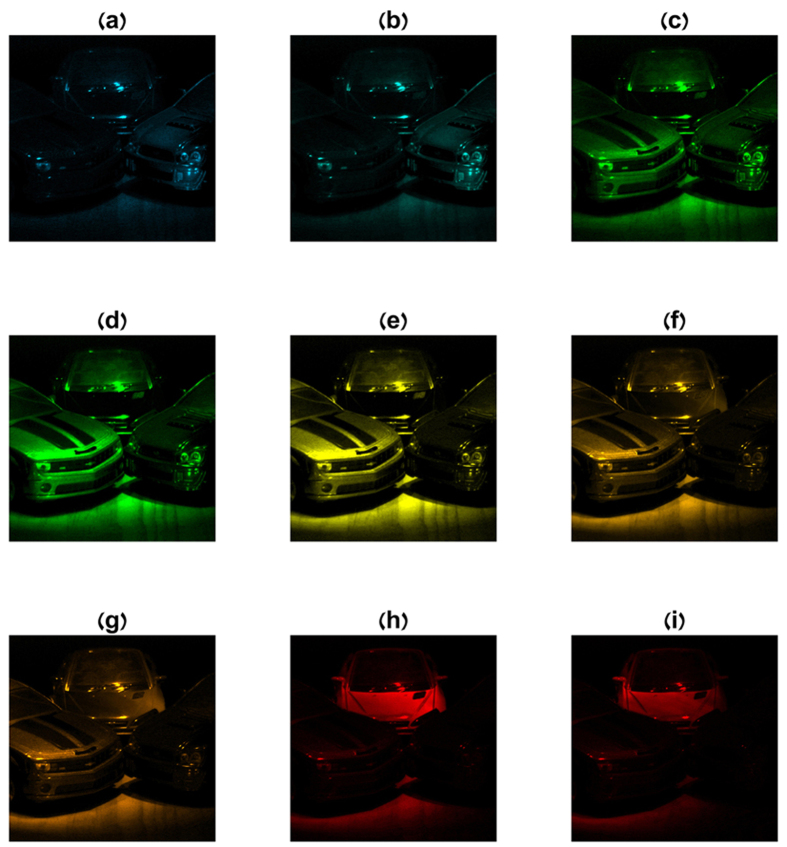
Ultra-spectral reconstruction at different wavelengths of a car model. An ultra-spectral cube of 1000 spectral bands was recovered from a set of 100 multiplexed images. Nine subfigures (**a–i**) from the entire cube are presented corresponding to different wavelengths (482, 490, 518, 536, 566, 588, 595, 630, 642 nm).

The imaging experiment and the reconstruction of Fig. 6c were performed by capturing 137 compressive images containing 1024 × 1280 pixels under settings of appropriate voltages over the LCC device. From the captured set, a window of 950 × 950 pixels was used in the reconstruction process. The ultra-spectral cube was restored with 1197 spectral bands. Figure 7 displays nine images from the reconstructed data cube at different wavelengths.

## Discussion

We have presented a CS ultra-spectral imaging technique based on a single variable LC retarder and a parallel sensor array. Experiments in emission and reflection configurations have demonstrated compressibility of an order of magnitude. Gigapixel spectroscopic images with ultra-spectral resolution were reconstructed using a scanning process that captured only ~10% of the number of samples that would have been required with a conventional system. The system can be built with a small geometrical form owing to the avoidance of spectral-to-spatial optical converting elements. The possibility of fabricating the spectral modulator integrated with the sensor provides high spectroscopic imaging performance in a very small volume.

Potential applications for miniature ultra-spectral imaging systems can be found in bio-medicine, spectroscopic microscopy, optical bio-sensing, precise agriculture, fabrication process monitoring and other fields that need compact, accurate and fast imagers with high spectral resolution. The use of CS-MUSI in those applications could provide a shorter scanning time and higher imaging quality for the same scanning effort.

The system can be implemented by means of spectral encoding of the received light, as demonstrated above, or, alternatively, by using the same LCC for spectrally structured illumination. The former scheme is more appropriate for remote sensing, whereas the latter is more suited to spectroscopic microscopy applications. The key element in the sensing process is the LCC that performs a form of spectral harmonic encoding, which is one of the preferred CS encoding methods^6^,^7^. All the pixels are spectrally encoded in parallel in the same manner; therefore, the overall sensing process can be represented by separable spatial and spectral operators. As a result, the image processing can be carried out efficiently with sparse structured block matrices^27^,^28^. Moreover, since only the spectral domain is encoded, there is no loss of spatial information. Another feature of the technique is its robustness to pixel saturation phenomena that may be caused by high illumination conditions. In many practical cases, only a subset of the total spectral modulation leads to saturation and only at certain spatial locations. In such a case, these saturated measurements can be discarded in the reconstruction process, resulting in only partial spectral information loss with negligible spatial loss. This flexibility, in terms of using selected measurements in the reconstruction process, can be exploited to cope with interference noise or with occlusions in a sequence of images.

It should be mentioned that the proposed technique has two limitations. Although the proposed technique allows faster spectral scanning due to the low number of snapshots, there is a limit on the acquisition frame rate, due to the time delay between consecutive shots caused by the finite LCC time response. Generally, the response time for the LCC is a function of the cell thickness, and the response time is thus longer for thicker cells. This limitation can be reduced by operating the cell in its transition state or with specially designed electronic functions, or by using faster LC structures such as ferroelectric LCs. The second limitation of the technique is the requirement of large computational resources to store and process the data. Nevertheless, the computational running time can be significantly reduced by using separable matrix operation with large scale parallel computing. Another solution for this can be found by the parallel processing of spatial blocks using GPU or multi-core CPU systems.

A property of the proposed system that carries both advantages and a disadvantage is the fact that the spatial domain is not encoded. From the CS point of view this is a disadvantage because the compression is lower than could have been potentially obtained with the encoding of all the three domains. On the other hand, the lack of spatial encoding allows parallel processing and facilitates the spectral imaging of moving objects. Since the spatial information is unaffected, moving objects can be treated as a sequence of images using standard spatial processing tools developed for regular videos. A second advantage is the improved robustness to loss of frames during the scanning, and robustness to temporal occlusion.

In conclusion, we have presented and demonstrated a novel (CS-MUSI) technique and system. Thanks to CS, we have demonstrated an order of magnitude shorter acquisition time, compared to that which would be required by an equivalent conventional system. For the reconstruction of ultra-spectral data cubes of 1.3 gigavoxels only 100 exposures of ~1 Mpixel were taken, requiring about 100 Mbytes; this is around ten times less than would be required with a conventional approach. The system was demonstrated for spectral imaging in the visible regime, but the technique can also be adapted to other regimes of the spectrum.

## Methods

### The compressive sensing model of the system

As explained above, the CS-MUSI technique provides a framework for the reconstruction of an *n* dimensional vector **f** from *m* dimensional linear measurements


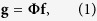


where *m* < *n* and **Φ** is the *m* × *n* sensing matrix. The underlying assumption is that **f** is sparse, or has some sparse representation in some transformed domain. The reconstruction is carried out by an *l*_1_ minimization technique^29^. To define the sensing model in our context, let 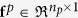 denote a lexicographic form of a regular grayscale image with a size of *n*_*p*_ = *n*_*x*_ × *n*_*y*_ pixels. To represent the full 3D spectroscopic imaging data we add to each pixel *i* = 1, .., *n*_*p*_, its spectroscopic information, by replacing each entry in **f**^*p*^ with a vector 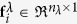 representing the *n*_*λ*_ spectral band distribution. As the CS-MUSI encodes only the spectral information without spatial encoding^21^, the sensing matrix **Φ** in equation (1) has the form of:


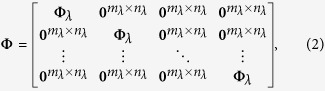


where the matrix **Φ**_*λ*_ represents the spectral modulation of each pixel. The size of **Φ**_*λ*_ is *m*_*λ*_ × *n*_*λ*_, with *m*_*λ*_ < *n*_*λ*_^17^. **Φ**_*λ*_ is determined by the LCC spectral encoding matrix. Its *k*, *l*^th^ entry is given by^30^:


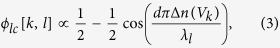


where Δ*n*(*V*_*k*_) is the birefringence at LCC voltage *V*_*k*_, *d* is the geometric path in the LC solution and λ is the wavelength. Equation (3) holds for polarizers with perpendicular orientation, where the plate’s optic axis is oriented at 45° to the polarizer axis. The operational assumption for CS-MUSI is that the reflected or emitted light from imaged objects is totally un-polarized.

Equation (2) is in the form of a block matrix and can be written in an abbreviated form as **Φ** = **I**⊗**Φ**_*λ*_, where the operator ⊗ denotes the Kronecker tensor product^28^,^31^ and **I** is the identity matrix of size *n*_*p*_ × *n*_*p*_. As shown in^27^,^28^, the Kronecker tensor products of CS matrices are very efficient in practice, despite not being optimal universal CS sensors. By using Kronecker tensor product properties^32^,^33^, a block sensing matrix, **Φ**, can be presented mathematically as:


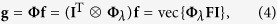


where 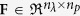 is a matrix form of the spatial spectral *3D* hypercube. The structure of **F** is given by the rearrangement of the spectral information of each voxel in the cube in a lexicographic order. Equation (4) provides a simple way to handle the huge matrix vector multiplication of equation (1) in the case of ultra-spectral imaging. Owing to the separable sensing mechanism of the system, parallel processing is natural. Parallel processing can be performed by employing multi-thread on a multi-core CPU or GPU.

### Experimental system setup

**Figure 8:**
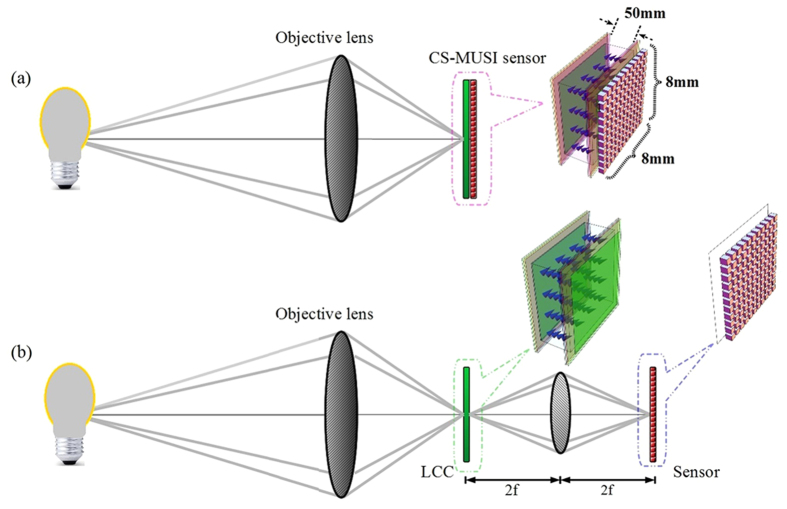
CS-MUSI and experimental setup. (**a**) The CS-MUSI system contains an objective lens and the CS-MUSI sensor described in Fig. 1; (**b**) The equivalent experimental setup. In the experimental setup, the objective lens forms an image on the LCC and the relay lens maps the image 1:1 on the sensor array.

The CS-MUSI system is illustrated in Fig. 8a. In this system an objective lens is forming an image on the sensor (Fig. 1). The experimental optical setup is slightly differed from the one presented in Fig. 8a, but is optically equivalent. In the reported experiment (Fig. 8b), the imaging sensor and the LCC were physically separated but were optically conjugated through a single lens imaging system with unit magnification. The LCC was placed in the image plane of an objective lens. The light transmitted through the LCC was conjugated to a sensor array using a second single 1:1 relay lens. The optical sensor was a uEye CMOS UI-3240CP-C-HQ with 1280 × 1024 pixels with a pixel size of 5.3 μm × 5.3 μm with 8-bit grayscale level radiometric sampling. The LCC was placed in between the two linear polarizers and had a diameter of 58 mm. The first polarizer was oriented perpendicular to the second and at an angle of 45-degrees with respect to the optic axis of the LCC. The LCC was manufactured in-house for this experiment, with a cell gap of approximately 50 μm and clear aperture of about 8 mm × 8 mm, obtained by inserting glass spacers at the corners of the ITO coated glass plates. The alignment was achieved using photosenstivie polymer layers (from Rolic-Switzerland) spun on top of the ITO and irradiated with linearly polarized ultraviolet light. The plates were arranged in an anti-parallel geometry and a nematic E44 mixture (Merck) filled the gap by capillary action. The electronic setup consists of a computer that controls the function generator that was synchronized to the electro optical sensor. The LCC was driven using a function generator with a set of sine waveform voltages of different amplitudes in the range 0 ~ 10000 mV. The function generator signals had 16-bit precision and its output fed an analog amplifier that drove the LCC. The closed loop control of the measurement was made by using a 12-bit, 100 kS/s Measurement Computing Data Acquisition USB DAQ Device.

### Calibration

The reconstruction process requires precise specification of the spectral encoding. The theoretical expression in equation (3) cannot be applied directly because the dependence of the birefringence on the voltage and the material dispersion are unknown. Therefore a calibration process was performed, in which the spectral responses of the LCC and of the entire system were measured as a function of the LCC voltage. Using a point light source with measured power spectral density, the system’s spectral transmission response was found with a high precision grating spectrometer. The overall system spectral transmission was measured by placing a spectrometer instead of the CMOS sensor. We performed a systematic calibration process for LCC voltages in the range 0 ~ 10000 mV with steps of Δ*V* ~ 2mV. Figure 2a presents the system spectral response map that was used as a reference from which the appropriate rows for a specific imaging process were used, for example Fig. 2b.

## Additional Information

**How to cite this article**: August, I. *et al*. Miniature Compressive Ultra-spectral Imaging System Utilizing a Single Liquid Crystal Phase Retarder. *Sci. Rep*. **6**, 23524; doi: 10.1038/srep23524 (2016).
